# MYC as a key target for melanoma therapy

**DOI:** 10.3389/fcell.2026.1750941

**Published:** 2026-08-04

**Authors:** Manuel Lillo-Valero, Mariano F. Zacarías-Fluck, Laura Soucek

**Affiliations:** 1 Models of Cancer Therapies Group, Vall d’Hebron Institute of Oncology (VHIO), Instituto de Investigación Sanitaria Hospital Universitari Vall d’Hebron (IIS IR-HUVH), Vall d’Hebron Barcelona Hospital Campus, Barcelona, Spain; 2 Peptomyc S.L., Vall D’Hebron Barcelona Hospital Campus, Barcelona, Spain; 3 Department of Biochemistry and Molecular Biology, Universitat Autònoma de Barcelona, Bellaterra, Spain; 4 Institució Catalana de Recerca I Estudis Avançats (ICREA), Barcelona, Spain

**Keywords:** immune therapy, melanoma, MYC, MYC inhibition, resistance, targeted therapy

## Abstract

Melanoma, the most aggressive form of skin cancer, arises from malignantly transformed melanocytes. Its incidence and mortality are increasing despite the enormous advances in both the knowledge of the disease and the development of effective therapies. From a genetic point of view, cutaneous melanoma can be classified as driven by mutations in *BRAF*, *NRAS* or *NF1*, or the absence of these mutations (triple wildtype). *MYC*, an oncogene deregulated in most human cancers, drives tumor progression by exerting a broad array of functions that affect all the hallmarks of cancer. In this context, increased MYC activity in melanoma patients occurs by amplification of its gene locus, or by upstream signaling leading to its stabilization and overexpression. In this review, we discuss the role of MYC in melanoma, its relationship with the response and resistance to both targeted therapies and immunotherapy, the different approaches that have been tested to target MYC in this disease, and finally, the future directions towards MYC becoming a target in melanoma.

## Overview of cutaneous melanoma

Melanoma arises from the malignant transformation of melanocytes, specialized cells derived from neural crest stem cells that produce the pigment melanin (Centeno et al., 2023). This transformation can occur not only in the skin but also in the eye, giving rise to uveal melanoma (Chattopadhyay et al., 2016), and in glabrous skin of the palms, soles and the nail unit, giving rise to acral melanoma (Basurto-Lozada et al., 2021). However, the most frequent form of this type of cancer is its cutaneous form, which is highly associated with environmental factors, such as exposure to ultraviolet (UV) radiation, excessive tanning and sunburns, geographical location and the use of pesticides (Strashilov and Yordanov, 2021).

According to the latest data published, the number of new cases in 2021 was ∼300,000 for malignant melanoma, ∼4,400,000 for basal cell carcinoma (BCC), and ∼1,900,000 for squamous cell carcinoma (SCC), which correspond to incidences of 4.56%, 66.82%, and 28.61%, respectively. However, malignant melanoma accounted for more than half of the disability-adjusted life years (DALYs) (58.06%), reflecting its higher mortality burden compared to non-melanoma skin cancers, followed by SCC (41.87%) and BCC (0.07%) (Zhou et al., 2025). While incidence continues to rise, especially among older adults, in recent years mortality rates have declined in high-income countries, where survival rates have improved, with 5-year survival now exceeding 94% in the US upon early detection (Didier et al., 2024).

This improvement in survival is largely attributed to significant advances in both diagnosis and treatment (Wang et al., 2025). Currently, management strategies are highly stage-dependent and continue to evolve due to progress in surgical techniques, molecular diagnostics, and the development of targeted therapies and immunotherapies (Lopes et al., 2022). For patients with early-stage disease (*in situ* or localized melanoma), surgical excision remains the treatment of choice and is curative in virtually all cases, underscoring the importance of early detection in this tumor type (Lopes et al., 2022; Wang et al., 2025).

In contrast, patients with more advanced disease, unresectable or metastatic stage III or stage IV melanoma, are now treated with immune checkpoint inhibitors (ICIs), such as pembrolizumab, nivolumab, and ipilimumab-based regimens (Larkin et al., 2019).

In the context of personalized medicine, patients whose melanomas harbor *BRAF* mutations can also benefit from targeted combination therapy with BRAF (vemurafenib, dabrafenib, encorafenib) and MEK inhibitors (cobimetinib, trametinib, binimetinib) (Lopes et al., 2022).

Overall, though, melanoma remains the most lethal form of skin cancer due to its high invasiveness and strong metastatic potential in its most advanced stages, particularly to the brain, where effective therapeutic options are still limited and survival outcomes remain poor.

## From melanocytes to malignant melanoma

Melanocytes reside primarily in the basal epidermis and hair follicles, where they respond to UV-induced DNA damage by producing melanin through MSH–MC1R signaling, providing essential photoprotection (Abdel-Malek et al., 2008). This mechanism is evident in populations of European ancestry, in which low melanin production in the skin - which evolved as a genetic adaptive trait to chronic low UV radiation exposure over generations - represents the primary risk factor for malignant melanoma (You et al., 2022). Tumors arising on chronically sun-exposed skin typically develop in older individuals (>55 years old), occur on anatomical sites such as head and neck, are associated with higher burden of NF1 and ROS1 mutations, and exhibit significantly higher number of pathogenic mutations, but no differences in the number of UV-induced mutations (Millán-Esteban et al., 2021). Conversely, melanomas associated with intermittent sun exposure are more common in younger individuals, arise on the trunk or proximal extremities, are frequently driven by *BRAF* ^*V600E*^ (Maldonado et al., 2003), and show comparatively median to lower mutational loads compared to the NF1 subtype (Mao et al., 2026).

Melanoma initiation and progression is a highly heterogeneous and multi-stage evolutionary process that challenges traditional models of uniform transformation from benign nevi to malignant disease (Centeno et al., 2023). Although *BRAF* mutations are observed in up to 80% of benign melanocytic nevi, these lesions rarely advance to melanoma due to robust oncogene-induced senescence and active immune surveillance mechanisms that constrain cellular proliferation (Damsky and Bosenberg, 2017; Schiferle et al., 2021). In the minority of cases where malignant transformation occurs, it typically involves the accumulation of additional genetic alterations, notably in genes such as *TERT* and *CDKN2A*, which serve as critical drivers of oncogenesis (Shain et al., 2015). Of note, MYC overexpression, at least *in vitro*, can bypass senescence, more prominently in BRAF^V600E^ melanomas (Zhuang et al., 2008). By contrast, melanomas associated with chronic UV exposure more commonly arise *de novo*, rather than from pre-existing nevi (Pellegrini et al., 2021). These divergent origins and mutational pathways underscore the distinct evolutionary trajectories and genomic landscapes that characterize melanoma pathogenesis.

## Molecular biology of melanoma

Exogenous mutagens, particularly UV radiation, are the primary contributors to the exceptionally high somatic tumor mutational burden (TMB) observed in melanomas (Dousset et al., 2021). Chronic exposure to UV radiation induces extensive DNA damage, leading to the accumulation of numerous genomic mutations within melanocytes (Pfeifer, 2020).

**FIGURE 1:**
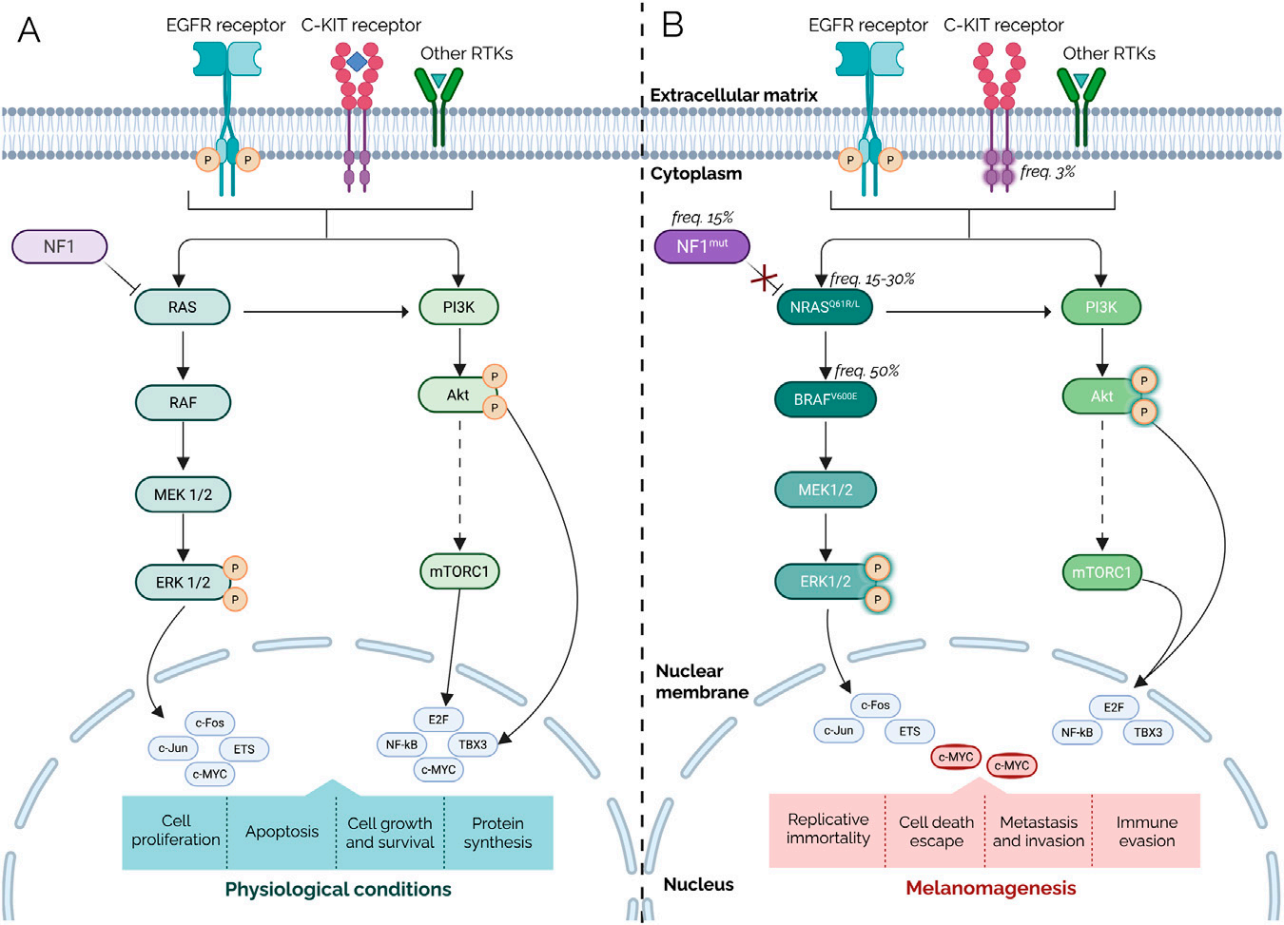
Key signaling pathways and effectors underlying malignant melanoma transformation**.** Under physiological conditions **(A)**, the MAPK and PI3K-Akt pathways are tightly regulated and coordinate essential cellular processes, including growth, proliferation, and differentiation. Conversely, in melanoma **(B)**, recurrent driver mutations disrupt this regulation, leading to sustained hyperactivation of both pathways. In addition to modulating their principal downstream transcriptional programs, MAPK and PI3-Akt signaling converge on the activation of c-MYC, a central oncogenic effector that promotes multiple cancer hallmarks, including replicative immortality, resistance to apoptosis, metastatic and invasive behavior, and mechanisms of immune evasion.

The most frequent somatic mutations found in both chronically and intermittently sun-exposed cutaneous melanomas typically affect genes involved in essential cellular processes such as proliferation and growth, metabolism, and resistance to apoptosis. In most cases, these alterations converge on the hyperactivation of key signaling cascades (Figure 1A), most notably the well-characterized Mitogen-Activated Protein Kinase (MAPK) signaling pathway (Davies et al., 2002), as well as the phosphoinositol-3-kinase (PI3K)–AKT pathway (Davies et al., 2009).

Indeed, a comprehensive study by The Cancer Genome Atlas (TCGA) program (Cancer Genome Atlas Network, 2015) classified melanomas into four major molecular subtypes based on their somatic mutational status or predominant driver mutation: 1) B-Raf proto-oncogene serine/threonine kinase mutant (*BRAF* ^*mut*^), 2) NRAS proto-oncogene GTPase mutant (*NRAS* ^*mut*^), 3) Neurofibromin 1 (*NF1* ^*mut*^), and 4) triple wildtype (WT) (Figure 1B). Remarkably, this classification underscores that up to 90% of melanomas exhibit aberrant activation of the MAPK signaling pathway, which serves as a central driver of tumor cell proliferation and survival.

Of all the aberrant mechanisms leading to excessive activation of the MAPK signaling pathway, mutations in the *BRAF* gene stand out, as they account for up to 50% of all cutaneous melanomas (reviewed in Ascierto et al., 2012). The most frequently detected alterations in this MAPK effector are missense mutations that result from the substitution of valine at codon 600 into glutamic acid (V600E). However, though less common (5%–12%), other amino acid substitutions, such as the replacement by lysine (V600K), have also been reported (Greaves et al., 2013).

*NRAS* mutations are found instead in approximately 15%–30% of melanomas and sustain abnormal activation of both the MAPK and PI3K pathways. Interestingly, although rare exceptions have been described, *BRAF* and *NRAS* mutations are generally considered mutually exclusive (Jenkins and Sullivan, 2016; Perfetto et al., 2025).

*NF1* is a tumor suppressor gene that acts as a negative regulator of the RAS protein family (Basu et al., 1992; O’Connell et al., 1992); thus, *NF1* loss-of-function mutations result in the hyperactivation of the RAS signaling cascade.

Finally, in triple WT melanomas (lacking mutations in *BRAF*, *NRAS*, and *NF1*), recurrent alterations can be found in genes such as *KIT* (encoding a receptor tyrosine kinase), the *TERT* promoter, *CDKN2A*, and *PTEN* (Strashilov and Yordanov, 2021).

## Current treatment landscape in melanoma patients

Advances in our understanding of the key molecular aspects of melanomagenesis have transformed the therapeutic landscape of melanoma, enabling the development of more precise and personalized therapies that inhibit specific oncogenic pathways, as well as immunotherapeutic approaches (Castellani et al., 2023). Consequently, the clinical management of melanoma has undergone a major shift toward integrative, mechanism-based strategies that have significantly improved patient survival, especially in advanced-stage disease.

The use of targeted therapies for the treatment of advanced melanoma harboring *BRAF* ^*V600E*^ mutation began in 2011, when the Food and Drug Administration (FDA) granted approval for Vemurafenib, the first selective inhibitor of this mutation (Kim et al., 2014), followed by its approval by the European Medicines Agency (EMA) in 2012 (EMA, 2012). In the pivotal BRIM-3 trial, vemurafenib significantly outperformed standard chemotherapy (dacarbazine), achieving a median progression-free survival (PFS) of 5.3 months versus 1.6 months, and a 6-month overall survival (OS) rate of 84% compared with 64% in the chemotherapy arm (Chapman et al., 2011; Ravnan and Matalka, 2012).

However, despite initial clinical benefit, patients frequently acquire resistance to BRAFi within 6–7 months (Sosman et al., 2012). This prompted the development of combination strategies targeting downstream components of the MAPK pathway. In 2014, Trametinib became the first MEK inhibitor used in combination with the BRAFi Dabrafenib, achieving more sustained MAPK suppression and yielding a median progression-free survival (PFS) of 11.4 months and a 34% 5-year OS (Eroglu and Ribas, 2016; Long et al., 2016).

Although this combination as first-line therapy for *BRAF* ^*V600E*^-mutated melanoma has markedly improved survival outcomes (Robert et al., 2019), the emergence of immune checkpoint inhibitors has been even more groundbreaking, since immunotherapy offers durable clinical benefits across a broader range of patients, including those bearing non-BRAF-mutated melanomas (Noringriis et al., 2025). The first immune checkpoint inhibitor (ICI), ipilimumab, was approved in 2011 for unresectable or metastatic melanoma. Indeed, a Phase III trial in 2010 demonstrated that this anti-CTLA-4 antibody extended median OS to 10.1 months, compared with 6.4 months in the control group treated with a glycoprotein 100 (gp100) peptide vaccine (Hodi et al., 2010). Long-term analyses revealed a 3-year survival plateau of ∼22% and 7-year survival of ∼17% across nearly 1.900 patients (Schadendorf et al., 2015). Nonetheless, its overall efficacy as monotherapy was limited.

This limitation was addressed with the development of anti-PD-1 antibodies, such as pembrolizumab and nivolumab, and with combinatorial regimens integrating CTLA-4 and PD-1 blockade (Gellrich et al., 2020). In the landmark CheckMate-067 phase III trial (Larkin et al., 2015), the median progression-free survival was 11.5 months with nivolumab plus ipilimumab, compared with 2.9 months with ipilimumab and 6.9 months with nivolumab, whereas in patients with PD-L1-positive melanomas, the PFS was 16 months for either the combination or nivolumab alone. A remarkable 10-year follow-up study then reported a median OS of 71.9 months with nivolumab plus ipilimumab, 36.9 months with nivolumab, and 19.9 months with ipilimumab alone (Wolchok et al., 2025), firmly establishing combination immunotherapy as one of the most effective treatments for advanced melanoma.

Matching-adjusted indirect comparisons were then conducted between nivolumab + ipilimumab (Check-Mate 067/069 studies) and BRAF + MEK inhibitors (COMBI-d, COMBI-v and coBRIM studies). These studies showed that Nivolumab + ipilimumab significantly improved clinical outcomes with respect to BRAFi + MEKi, with benefits emerging after 1 year (Atkins et al., 2019).

While these advances are impressive and encouraging, acquired resistance to standard-of-care treatments remains a substantial clinical challenge in melanoma. As we will see in the following sections, MYC has emerged as a convergent downstream mediator activated across diverse resistance pathways.

## MYC structure, function and regulation

The MYC family comprises c-MYC, MYCN and MYCL. They have a N-terminal transactivation domain (TAD) containing highly conserved regulatory elements called MYC boxes that interact with hundreds of proteins regulating chromatin remodeling, transcription and MYC stability, a central region, and a C-terminal basic region helix-loop-helix leucine zipper (bHLHLZ) domain, which mediates dimerization with MYC obligate partner MAX and the binding to DNA (Beaulieu et al., 2020).

C-MYC, from now on MYC, is the archetype of an intrinsically disordered protein, displaying molten-globule like behavior. It is not a pure random coiled protein, but a combination of loose cores with persistent secondary structure elements that lack precise packing (Beaulieu et al., 2020). MYC alone cannot form dimers and bind to DNA, but when it forms heterodimers with MAX and binds DNA, it adopts a more globular fold (Nair and Burley, 2003). In this form, it preferentially binds to the canonical CACGTG E-boxes, although it can also bind non-canonical ones with lower affinity (CANNTG) (Allevato et al., 2017).

MYC levels are normally tightly regulated primarily by post-translational modifications. Stepwise phosphorylation of S62 by ERK family kinases, followed by phosphorylation of T58 by GSK3β and subsequent dephosphorylation of S62 by PP2A and PIN1, promotes MYC degradation, a process in which MYC becomes the substrate of E3-ligases, getting ubiquitinated prior to its final degradation (Thomas and Tansey, 2011).

MYC acts as a transcription factor and is involved in the regulation of a plethora of physiological and pathological processes. The most prominent ones are proliferation, differentiation, ribosome biogenesis, protein translation, metabolism, and development. When the regulation of MYC activity is lost, however, MYC may impinge on and contribute to all the Hallmarks of Cancer (Dhanasekaran et al., 2022).

## MYC status in cutaneous melanoma

Several studies characterized the expression level of MYC and its relationship with melanoma aggressiveness in cell lines and patient samples. Early studies focused on copy number status and expression level. MYC, along with K-Ras, N-MYC, and PDGFB, was initially found to be aberrantly overexpressed in a panel of 44 fresh samples from sarcoma and melanoma (Shin et al., 1987). Three years later, MYC mRNA level, among 12 other oncogenes, was found to be increased in 2 cell lines established from metastatic melanoma, when compared to melanocyte cell lines (Husain et al., 1990). Then, MYC relevance in tumor staging was described by Lazaris and colleagues, who showed that, in a series of 60 malignant melanomas, immunohistochemical expression of MYC correlated with high mitotic rate and advanced Clark’s level, the historical melanoma staging system (Lazaris et al., 1995). Subsequent analysis of 94 melanoma primary tumors and metastases showed a significant negative correlation between the expression of MYC and cell surface antigens as assessed by flow cytometry, providing the first evidence of a link between MYC and immune evasion (Grover et al., 1996).

In line with these initial observations, a prospective study of 50 ethanol-fixed melanoma samples, including tumors, regional, and distant metastases, showed that high expression of MYC predicted poor outcome (Ross and Wilson, 1998). The value of nuclear MYC expression level as an independent prognostic marker was then confirmed in a study of 97 head and neck melanomas (Chana et al., 2001), and by another one by flow cytometry in 92 primary melanomas where MYC expression alone was used as a more accurate independent prognostic marker than Breslow depth (Grover et al., 2003). At the preclinical level, manipulation of MYC expression in IGR39D melanoma cells showed the impact of MYC on cell growth rate, chemotaxis, anchorage-independent growth, and tumor growth *in vivo* (Schlagbauer-Wadl et al., 1999).

On the other hand, the first evidence of MYC amplification in cutaneous malignant melanoma was described in 2001, when copy number gains in 8q24, harboring the MYC locus, were shown in non-chronically sun-damaged skin, and were associated with amelanotic lesions, aggressive clinical course and visceral metastases (Kraehn et al., 2001). Later studies reported MYC copy number alteration in 41% of melanoma patients (Moore et al., 2008) and a correlation between 8q24 amplification and increased MYC expression levels, with concomitant reduction in the levels of melanocyte inducing transcription factor (MITF) and tyrosinase (TYR), an enzyme involved in melanin production (Pouryazdanparast et al., 2012).

In addition, RT-PCR analysis showed that co-expression of *MYC* and *BCL2*, which prevents MYC-induced apoptosis, is found in 83% of melanoma metastases, in contrast to 35% of primary melanomas and melanocytic nevi (Utikal et al., 2002).

## MYC in acral and mucosal melanoma

Although acral, mucosal, and cutaneous melanomas arise in different locations, they all depend on the same core signaling pathways. Several studies in acral melanoma (AM), for example, have identified amplifications in genomic regions containing genes involved in signaling pathways (MAP2K1, GAB2, PAK1), cell cycle control (CCND1, CDK4, MDM2), and transcriptional co-activators (EP300). Interestingly, AM has a different profile of driver mutations than CM: 26% in NF1, 21.8% in BRAF, and 10% in NRAS. 32.2% are triple wildtype whereas other significant mutations identified in AM included TYRP1, PTEN and c-KIT (Newell et al., 2022; Carvalho et al., 2023).

A meta-analysis of 147 acral and 93 mucosal melanomas identified 16 significantly mutated genes, 13 of which are known melanoma drivers that activate MAPK or other pathways (Wang et al., 2022). An analysis of 101 cutaneous (CM), 28 acral, and 27 mucosal melanomas (A/M) showed that MYC, RUNX1T1, CDK4, and CRKL amplifications were present in at least 10% of A/M melanomas while being uncommon to CM (Turner et al., 2024).

Hence, even in these two other melanoma subtypes, MYC would be activated by key mediators such as MAPK, PI3K, p16, p53, and telomere maintenance, in accordance with its role as an intracellular signaling hub (Dang, 2012).

## MYC role in acquiring resistance to standard-of-care melanoma therapies

### MYC and targeted therapies resistance

Beyond being a major driver of tumorigenesis, MYC also has a clear role in therapy resistance (Donati and Amati, 2022). In 2017, Singleton and colleagues performed a bioinformatic analysis of a series of melanoma patient samples and identified MYC as the top hit among potential convergent effectors of resistance (Singleton et al., 2017). Indeed, in this study, the authors focused on genes and pathways that were suppressed following drug treatment but rebound at relapse, and found that MYC had greater than a 90% of probability of expression rebound in approximately 80% of resistant tumors, and more than 50% of rebound in all tumors, where resistance was driven by a range of mechanisms, including activation of the ERK, PI3K, and Notch1 pathways. Further experiments confirmed that MYC activation was both necessary and sufficient to generate resistance to BRAF inhibitors and that major pathways of MAPK resistance converged on MYC (Singleton et al., 2017). Another mechanism of resistance to BRAFi and BRAFi/MEKi involves ABL1/2 kinases expression, which again results in the activation of ERK and MYC (Tripathi et al., 2020). Hence, combination therapies that simultaneously target BRAF, MEK, and MYC in BRAF-mutant melanoma cells would have the unique property of selecting against resistance to these targeted inhibitors.

Further searches for vulnerabilities in treatment-resistant melanoma included CRISPR-Cas9 screens. One of them was performed on vemurafenib-resistant A375VR cells and identified the polyamine biosynthesis enzyme AMD1 (S-adenosylmethionine decarboxylase 1) as a druggable target whose inhibition reduces vemurafenib resistance. Importantly, sustained MYC activity in A375VR cells was responsible for increased AMD1, and either MYC or AMD1 inhibition reversed vemurafenib resistance (Park et al., 2024).

Very recently, Zhang et al. proposed a BRN2:MYC transcriptional axis that regulates the interconversion between therapy-resistant and tumorigenic phenotypes in melanoma. According to this report, melanoma cells exist in two mutually exclusive transcriptomic states: a MYC-driven proliferative one, and a dedifferentiated, invasive BRN2-high state enriched with therapy-resistant cells that are not directly tumorigenic *per se*. Transition between the two is through an intermediate, differentiated state and can be bidirectional. Hence, the equilibrium between them and the outcome towards tumorigenicity or unresponsiveness to treatment likely depends on cell intrinsic and extrinsic factors (Zhang et al., 2025).

### MYC and immunotherapy resistance

In the context of immunotherapeutic strategies in the treatment of melanoma, MYC also plays a pivotal role in shaping both treatment efficacy and development of resistance (Han et al., 2019). Indeed, MYC appears to influence tumor immunogenicity through the establishment of an immune-privileged tumor microenvironment, thereby modulating antitumor immune responses and contributing to therapeutic failure. One such mechanism involves the regulation of key immune checkpoint molecules (Casey et al., 2018; Nirala and Yustein, 2025). For instance, PD-L1 has been identified as one of the critical downstream targets of MYC: by directly binding to PDL1 promoter, MYC drives PD-L1 transcriptional activation, ultimately leading to T-cell exhaustion.

Another resistance mechanism of particular relevance in melanoma is the MYC-mediated suppression of antigen presentation, a process that is essential for cytotoxic T lymphocyte (CTL)–mediated tumor cell recognition and elimination. Specifically, MYC overexpression has been shown to reduce the expression of MHC class I molecules, along with multiple components involved in the antigen-processing machinery. Importantly, MYC-driven MHC-I downregulation correlates with reduced tumor immunogenicity and accelerates tumor progression (Versteeg et al., 1988).

In addition, MYC overexpression in melanoma cells leads to a reduction in responsiveness to IFNɣ through JAK2 downregulation, one of the kinases required for IFNƔR (IFNɣ receptor) dimerization. Silencing of either MYC or its obligate partner MAX by siRNA increases JAK2 expression and IFNɣ response, leading to a significant enhancement of the effector functions of T cells (Markovits et al., 2023).

Beyond exerting cell-intrinsic effects on antigen presentation and IFNγ signaling, MYC also acts as a master regulator of the tumor immune microenvironment by directly reprogramming the tumor secretome into a more suppressive one (González-Larreategui et al., 2026). In particular, MYC has been shown to trigger immunosuppression through secretion of inhibitory cytokines, such as IL-6, IL-10 and TGFß among others, that recruit Myeloid-Derived Suppressor Cells (MDSCs) and regulatory T cells (Tregs) (Kortlever et al., 2017; Sarkar et al., 2021). In parallel, MYC activation can polarize tumor-associated macrophages (TAMs) toward a M2-like phenotype by stimulating the secretion of pro-tumorigenic signals, such as NRF2, CCL9 or IL-13 (Feng et al., 2018; Schaer et al., 2025), which promote tumor growth and angiogenesis, and suppress antitumor immunity by inhibiting CD8^+^ T cells. Moreover, MYC dysregulation in tumor cells promotes fibroblast-mediated fibrosis and stromal remodeling, processes that are known to constrain immune cell access to the tumor niche (Baudino et al., 2002). Even though there is emerging evidence showing that MYC can modulate immunogenicity and treatment response in melanoma, dedicated mechanistic studies dissecting its effects in the tumor immune microenvironment of this disease are still scarce, especially compared with other tumor types.

At the clinical level, transcriptomic analyses of human melanoma samples refractory to anti-PD-1 and anti-CTLA-4 therapies reveal a marked enrichment of MYC-driven transcriptional programs. These signatures show strong associations with immune-excluded and dedifferentiated tumor phenotypes, as well as reduced cytotoxic T-cell infiltration (Lauss et al., 2024), underscoring MYC’s central role in modulating the immunologic landscape that dictates treatment outcomes.

Collectively, these observations establish MYC as a key orchestrator of both targeted therapies and immunotherapy resistance in melanoma. Through its pleiotropic activities, MYC compensates for upstream signaling pathway inhibition, diminishes tumor immunogenicity and promotes an immunosuppressive microenvironment (Li et al., 2023). Therapeutic strategies that integrate MYC blockade with SoC in melanoma therefore hold substantial promise for overcoming resistance, restoring effective antitumor immunity, and achieving durable clinical benefits.

## Targeting MYC in melanoma

**FIGURE 2:**
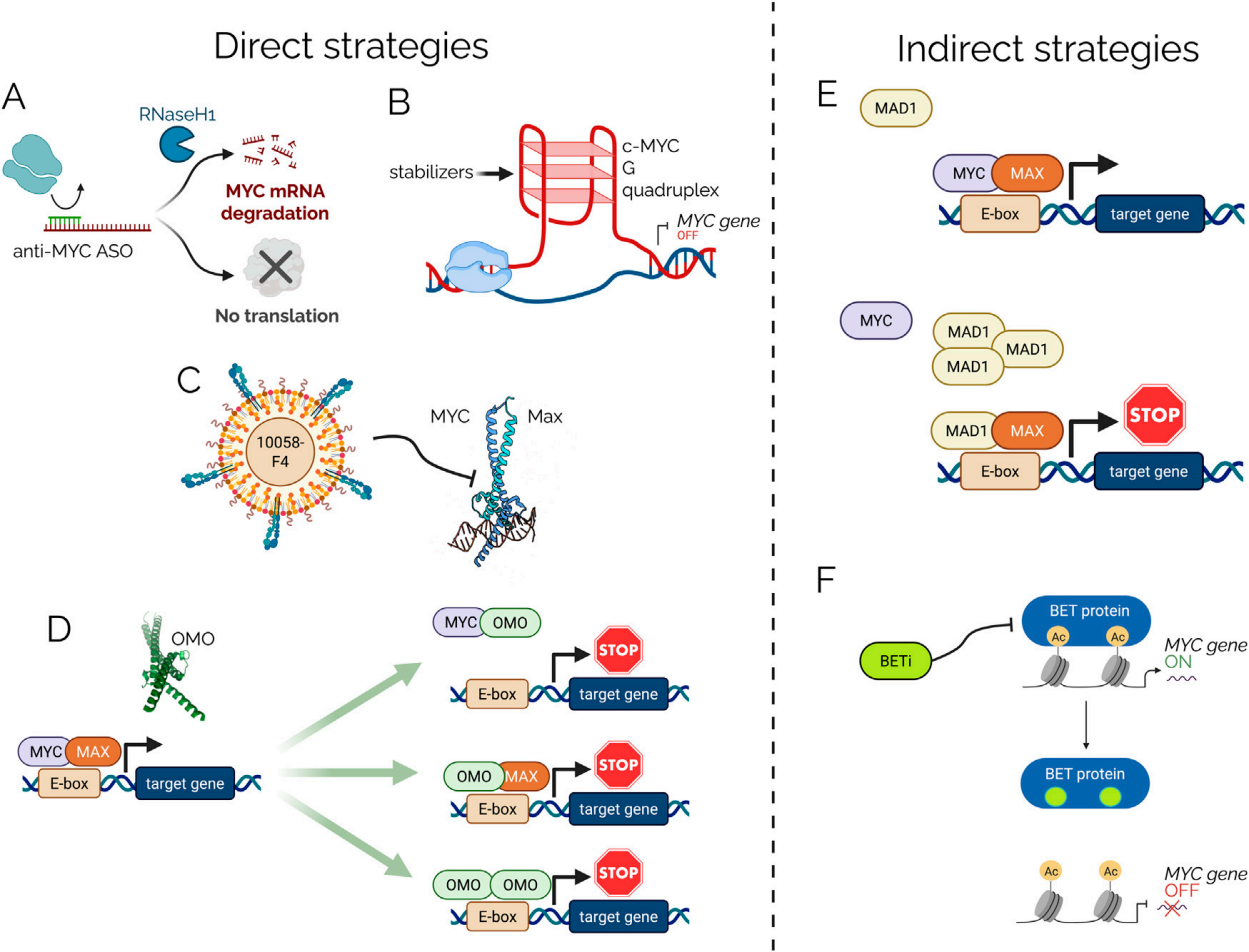
MYC-targeting strategies in melanoma and their clinical development status. Direct strategies **(A)** antisense oligonucleotides (ASO) against MYC target its mRNA for degradation and prevent protein translation (preclinical) **(B)** Stabilizers of the inhibitory G-quadruplex structure in MYC promoter decrease MYC transcription (Phase one) **(C)** Loading MYC inhibitor 10,058-F4 in a_v_b_3_-targeted polysorbate nanoparticles increases its potency and effectively inhibits the proliferation of melanoma cells (preclinical) **(D)** Omomyc prevents MYC-dependent transcription by sequestering MYC away from MAX and from DNA, and by forming heterodimers with MAX or homodimers that occupy the E-boxes shutting down gene transcription (Phase 2 CT). Indirect strategies **(E)** In tumor cells, MAX preferentially binds to MYC. Upon MAD1 overexpression, however, MAD1 replaces MYC in the heterodimers with MAX and binds DNA to block transcription (preclinical) **(F)** BET proteins facilitate MYC transcription. BETi bind to the acetyl-lysine recognition pockets of BET proteins, displacing them from acetylated histones, thus preventing the recruitment of the transcriptional machinery (Phase 2 CT).

MYC has long been one of the most sought-after targets in cancer therapy in cancer treatment, but it has been deemed undruggable until recently (Whitfield and Soucek, 2025). For what refers to melanoma, different approaches have been devised to target MYC, both directly and indirectly (Figure 2).

### Direct strategies

Almost 30 years ago, ribozyme-mediated gene targeting was employed to lower MYC mRNA expression, increasing melanoma cells’ doubling time, decreasing DNA synthesis and anchorage-independent growth (Ohta et al., 1996). Approximately at the same time, several pioneering studies focused on the use of antisense oligonucleotides (ASO) against MYC (Figure 2A). Inducible expression of anti-MYC ASO in BRAF-mutant M14 cells decreased MYC levels, arrested cells in G1 and increased apoptosis, with increased p27 levels (D’Agnano et al., 2001). MYC ASO also enhanced the efficacy of cisplatin *in vitro* and *in vivo* (Citro et al., 1998) and resensitized cisplatin-resistant cells, while also increasing the sensitivity of melanoma cells to radiation and doxorubicin treatment (Bucci et al., 2005; Pastorino et al., 2008). Finally, the encapsulation of MYC ASOs in lipid particles improved antitumoral efficacy *in vivo* against melanoma primary cultures (Leonetti et al., 2001). A similar approach using anti-MYC siRNA significantly reduced growth and increased apoptosis of B16F10 tumors through systemic or topical administration (Ruan et al., 2016).

Another inhibitor impacting MYC mRNA and protein stability in melanoma is BTYNB, which prevents IGF2BP1 binding to MYC mRNA, causing its destabilization and leading to MYC mRNA and protein downregulation. Consistently, the use of BTYNB *in vitro* in melanoma cells led to reduction in cell proliferation and inhibition of anchorage-independent growth (Mahapatra et al., 2017).

A different strategy exploits the transcriptional repression capabilities of the G-quadruplex (G4) structure in the MYC promoter (Figure 2B) (Siddiqui-Jain et al., 2002). In this context, the treatment with G4 stabilizer imidazole-benzothiazole conjugate IZTZ-1 effectively arrested B16F10 cells in G1 and increased their apoptosis *in vitro* and significantly delayed their tumor growth *in vivo* (Wu et al., 2020).

To date, though, the most common approach to inhibit MYC has been the disruption of its dimerization with MAX. For example, a_v_b_3_-targeted polysorbate nanoparticles containing the MYC inhibitor 10058-F4 were used to cause a four-fold decrease in the proliferation of C32 BRAF mutant human melanoma cells (Figure 2C) (Pan et al., 2015). In this same context, we recently demonstrated that the melanoma-restricted transgenic expression of Omomyc, a MYC dominant negative mini-protein able to sequester MYC away from MAX and from DNA (Massó-Vallés and Soucek, 2020), led to sustained tumor regression and inhibition of metastases independently of the driver mutation and TP53 status (Figure 2D) (Zacarías-Fluck et al., 2023). Through transcriptional analysis of Omomyc-expressing melanomas *in vivo*, we unveiled a gene signature that could identify melanoma patients with good and bad prognosis. Indeed, Omomyc-expressing melanomas bear striking similarities with melanoma patients with good prognosis, in terms of the expression of MYC, melanoma, and immune response related gene sets (Zacarías-Fluck et al., 2023).

### Indirect strategies

Other strategies to inhibit MYC in melanoma focused on the MYC extended network. One of its members is MXD1/MAD1, which acts as a MYC antagonist by preferentially binding to MAX (Figure 2E). *In vitro* perturbation of MYC activity by Mad1 overexpression through liposomal-mediated transfection in FEM cells increased their doubling time 2.8 times, with 50% inhibition of proliferation and accumulation in G0/G1, whereas *in vivo*, tumors that arose from Mad1-transfected clones were 4–5 times smaller in volume than their control counterparts (Ohta et al., 2002).

Another indirect approach to inhibit MYC is centered around the bromodomain and extra-terminal (BET) subfamily of human bromodomain proteins (BRD2, BRD3, BRD4) (Figure 2F). These proteins facilitate transcriptional activation (Rahman et al., 2011), with BRD4 being closely associated with MYC transcriptional activity. In melanoma, BRD4 is overexpressed in primary and metastatic tissues compared with melanocytes and nevi. Treatment with BETi caused MYC downregulation, impaired melanoma cell proliferation *in vitro,* and tumor growth and metastatic spread *in vivo* (Segura et al., 2013). Another approach with BETi involved its combination with ATR inhibitors (ATRi). ATR is a kinase activated by replication fork stalling by UV-induced DNA damage or by oncogenic replication stress (Lecona and Fernández-Capetillo, 2014). The combination of ATRi and BETi reduced tumor growth *in vivo* of A375 and B16F10 cells, and of a panel of PDXs (Muralidharan et al., 2017).

## Clinical implementation of MYC inhibition

The translational potential of MYC-targeting strategies is enormous. Most human cancers have increased MYC activity that sustains tumour growth and therapy resistance. Yet we still lack a clinically approved MYC inhibitor, and we have no defined biomarkers of MYC activity to select patients that could benefit the most from MYC inhibition. Indeed, simple MYC copy number increase seems not to be always predictive of MYC expression levels (Collinge et al., 2021; Kumon et al., 2025) and, in some cases, even MYC protein levels do not correspond to MYC transcriptional activity (Murphy et al., 2008; Richart et al., 2016). Hence, one could speculate that the only reliable predictive biomarker of sensitivity to a MYC inhibitor would be high MYC transcriptional activity. However, since MYC is an irreplaceable signalling hub for oncogenic signalling, it is also possible that even patients with low-MYC tumors could benefit from this therapeutic intervention.

Given all these technical and conceptual challenges, clinical approaches targeting MYC are scarce (Table 1) (Whitfield and Soucek, 2025). To the best of our knowledge, only three direct MYC-targeting drugs are currently in clinical trials: the selective small-molecule c-MYC degrader WBC100 (Xu et al., 2022), the small molecule IDP-121 (IDP Discovery Pharma, 2025) and OMO-103, the mini-protein therapeutic derived from Omomyc (Garralda et al., 2024). Besides them, indirect MYC targeting approaches include the BET bromodomain inhibitors ABBV-744 (Sheppard et al., 2020) and ZEN-3694 (Aggarwal et al., 2020), the CDK inhibitors SY-5609 (Hoffman-La Roche, 2026) and Dinaciclib (National Cancer Institute NCI, 2026), and the HDAC6 inhibitor ACY-1215 (Jennifer and Brown, 2026) which promotes c-MYC proteasomal degradation (Hey et al., 2022).

**TABLE 1:** Clinical development status of MYC-targeting compounds currently evaluated in clinical trials.

Drug	Indication	Clinical trial information
OMO-103	Advanced high-grade osteosarcoma	Phase 2 pilot. NCT06650514
OMO-103	Locally advanced or metastatic PDAC	Phase 1. NCT07089940
OMO-103	Treatment-naïve advanced metastatic PDAC	Phase 1b. NCT06059001
IDP-121	Relapsed/refractory hematologic malignancies	Phase 1. NCT05908409
WBC100	Relapsed/refractory acute myeloid leukemia	Phase 1. NCT07014449
ABBV-744	Myelofibrosis. alone or in combination with ruxolitinib or navitoclax	Phase 1b. NCT04454658
ZEN-3694	Metastatic castration-resistant prostate cancer, in combination with enzalutamide and pembrolizumab	Phase 2 with a safety lead-in cohort. NCT04471974

## Conclusion

MYC is a pivotal oncogene in melanoma, and its overexpression is associated with increased tumor aggressiveness and resistance to current therapies, highlighting MYC as a key therapeutic vulnerability in melanoma biology. Efforts to inhibit MYC, both directly and indirectly, have demonstrated substantial preclinical efficacy, including suppression of MYC-dependent transcriptional programs, attenuation of tumor cell proliferation, and reduced metastatic potential. Together, these findings provide strong mechanistic rationale and compelling justifications for advancing MYC-targeted interventions into a clinical melanoma setting.

In this context, therapeutic MYC blockade holds considerable promise not only to potentiate the efficacy of existing targeted and immunotherapeutic regimens, but also to overcome acquired resistance mechanisms that limit durable responses in advanced disease. Strategic integration of MYC-targeted agents with MAPK pathway inhibitors, immune checkpoint blockade, or other rational combinatorial approaches may represent a transformative avenue for achieving sustained tumor control.

Future research should prioritize rigorous clinical evaluation of MYC inhibitors and the identification of predictive biomarkers capable of guiding patient stratification and individualized treatment design. Collectively, these advances position MYC as a critical and actionable therapeutic target with the potential to reshape treatment paradigms for advanced and therapy-refractory melanoma in the coming years.
